# Multivariate Analysis of COVID-19 for Countries with Limited and Scarce Data: Examples from Nepal

**DOI:** 10.1155/2021/8813505

**Published:** 2021-01-27

**Authors:** Jyoti U. Devkota

**Affiliations:** Department of Mathematics, Kathmandu University, Dhulikhel, Nepal

## Abstract

This paper aims to understand the dynamics of the spread of COVID-19 for Nepal. It is carried out with the help of multivariate statistics techniques. Direct relationships among variables are obvious, as they are easily seen and measured. But, hidden variables and their interrelationships also have a significant effect on the spread of a pandemic. Multinomial logistic regression, odds ratio, linear mixed-effect models, and principal component analysis are used here to analyze these hidden variables and their interrelationships. Also, such studies are very important for countries with limited and scarce data. These countries do not have a backbone of good-quality official records. Understanding the spread of a disease in a developing country also helps in management and eradication of that disease. The multivariate daily data of new cases, deaths, recovered, total cases, total deaths, total recovered, and total infected (isolated) are used here. The daily incidence of new cases is also modeled here using nonlinear regression. Two best nonlinear models are discussed here. ARIMA models are used for analyzing and forecasting the progression of the variables for two months into the future. The impact of government restriction in the form of strict lockdown 1, partially relaxed lockdown 1, completely relaxed lockdown 1, and strict lockdown 2 is minutely analyzed. These controls were exercised to curtail the spread of the pandemic. The role of these controls in curbing the spread of the pandemic is also studied here. The results obtained from this study can be applied to other countries of South Asia and Africa.

## 1. Introduction

A novel coronavirus (COVID-19) originating initially in Wuhan, China, has spread to 213 countries. Up to 2 September 2020, there are 848255 deaths and 25,327,098 confirmed cases, worldwide [1]. The whole world, including Nepal, has taken special measures to control the disease. In Nepal, the first case of COVID-19 was reported on 23 January 2020. It was a 32-year-old Nepalese student who had recently returned from Hubei, China. The patient recovered, and contacts were also asymptomatic [2]. The Government of Nepal enforced a strict lockdown from 24 March 2020. But, this was eased partially on 11 June 2020. Limited public transportation was allowed to ply on the roads. Shops and restaurants were partially opened. The end of lockdown with just few restrictions was on 22 July 2020. With this ease in lockdown, the capital Kathmandu has become a hotspot of COVID-19. For example, on August 4, it had incidences of 62 new cases among nationwide 259 new cases. These numbers are much higher at current date; however, at that time, these figures were also the highest values. Due to a spike in the number of COVID-19 cases, a strict lockdown was again enforced on 19 August 2020 [3]. The highest incidence of new cases till that time for whole Nepal, amounting to 1016, was also on 19 August 2020. This also resulted in the steepest rise in the total confirmed cases to 28257. The surge in people entering Nepal from India had resulted in this rise of confirmed cases.

In this paper, a data-driven analysis of dynamics of evolution of COVID-19 in Nepal has been completed. Here, Nepal is taken as a model of a country from the developing world. The variables, namely, new cases, daily deaths, daily recovered, total cases, total recovered, total infected (isolated), and total deaths, are minutely analyzed. These variables are first explained by descriptive statistics and autoregressive integrated moving average (ARIMA) models. These ARIMA models are also used in forecasting the values of these variables two months into future. The behavior of incidence of new cases is also explained by two nonlinear regressions. The dynamics of change under the influence of complete lockdown, partially eased lockdown, end of lockdown with only few restrictions, and again, a strict lockdown is then modeled using categorical data analysis. Direct changes in these variables are obvious and can be measured directly. Here, the impact of latent variables and their interrelationships on COVID-19 cases have been studied. Linear mixed-effect models, multinomial logistic regression, and odds ratio are used to quantify this intensity of change. Principal component analysis is used to study the underlying interrelationship between the variables. It is also used in dimension reduction of the data. These analyses are conducted from the perspective of a country with limited and scarce data. Such countries lack good-quality official records needed to validate any collected data. Strong background of regular registration of vital events such as birth, death, and migration is also missing. Remote geographical locations and lack of awareness and incentives are the reasons behind this sorry state.

## 2. Materials and Methods

*Data*. Patient data are obtained from the COVID-19 updates official website of the Ministry of Health and Population, Government of Nepal [4]. The data are available from May 22, when the incidence of new cases was 50. Total deaths amounted to 3 and total cases to 507. This portal gives information on new cases, daily deaths, daily recovered, total cases, total recovered, total infected (isolated), and total deaths. The data from May 24 to September 2, 2020, are also analyzed from the perspective of two strict lockdowns and two phases in-between. But, there are some gaps in the data on daily recovered, total recovered, and total infected (isolated). Here, the missing data are interpolated using nonlinear regression and curve fitting techniques. The data on new cases, total cases, new deaths, and total deaths are also validated by the website of Open Data on COVID. Our World in Data [5] provides information on new cases, total cases, new deaths, and total deaths for many countries of the world including Nepal.

*Categorical Data.* Here, the variables under study are classified into several categories [6]. It is very suitable for cases where there is a lack of measurement instruments. In such cases, classification of data into several categories reduces the error and bias incorporated during data collection. The reason behind it is that overestimation and underestimation tend to cancel out each other. For Nepal's COVID-19 incidence, this technique of categorization is especially suitable for daily data of recovered, total recovered, and total infected (isolated).

*Multinomial Logistic Regression and Odds Ratio*. Multinomial logistic regression is used to predict the probability of category membership on a dependent variable based on multiple independent variables [7]. It is a simple extension of binary logistic regression that allows more than two categories of the dependent or outcome variable. Here, Y is a multinomial response variable. Here, the log odds of occurrence of variables of interest with respect to the reference category (here, the first) is described as follows:

(1) $$\begin{array}{c} l={\text{Log}}_{e}\left(\frac{y_{i}=k}{y_{i}=1}\right)=\beta_{0}+\beta_{1}x_{1}+\beta_{2}x_{2}+,\ldots ,+\beta_{k}x_{k}+\epsilon_{i}. \end{array}$$

Here, *l* is the log odds, *β*_*i*_ is the *i*^th^ coefficient of the equation, and *ϵ*_*i*_ is the error term. There is an odds ratio associated with each predictor. It is more than 1 in cases where predictors increase the logit, equal to 1 in cases where predictors do not have any influence on the logit, and less than 1 in cases where predictors decrease the logit. Here, log *it*=Log_*e*_((*y*_*i*_=*k*)/(*y*_*i*_=1)).

*ARIMA Model*. It stands for autoregressive integrated moving average model [8]. A nonseasonal ARIMA model is classified as ARIMA (*p*, *d*, *q*) model. Here, *p* is the number of autoregressive terms, *d* is the number of nonseasonal differences needed for stationarity, and *q* is the number of lagged forecast errors in the prediction equation.

In terms of *y*, the general forecasting equation is given by

(2) $$\begin{array}{c} \hat{y_{t}}=\mu +\varphi_{1}y_{t-1}+\varphi_{2}y_{t-2}+\varphi_{3}y_{t-3}+,\ldots ,+\varphi_{p}y_{t-p}-\theta_{1}e_{t-1}-\theta_{2}e_{t-2}-,\ldots ,-\theta_{q}e_{t-q},\ldots . \end{array}$$

*Linear Mixed-Effect Models.* Here, a dependent variable is modeled as a linear function of fixed and random effects [9]. It can be explained as follows:

(3) $$\begin{array}{c} y_{ij}=\beta_{1}x_{1ij}+\beta_{2}x_{2ij}+,\ldots ,+\beta_{n}x_{nij}+b_{i1}z_{1ij}+b_{i2}z_{2ij}+,\ldots ,+b_{in}z_{nij}+\epsilon_{ij}, \end{array}$$

where *y*_*ij*_ is the value of the outcome variable for a particular *ij* case, *β*_1_ through *β*_*n*_ are the fixed-effect coefficients, *x*_1*ij*_ through *x*_*nij*_ are the fixed-effect variables (predictors) for observation *j* in group *i* (usually, the first is reserved for the intercept/constant *x*_1*ij*_), *b*_*i*1_ through *b*_*in*_ are the random-effect coefficients which are assumed to be multivariate normally distributed, *z*_1*ij*_through *z*_*nij*_ are the random-effect variables (predictors), and *ϵ*_*ij*_ is the error for case *j* in group *i* where each group's error is assumed to be multivariate normally distributed.

*Principle Component Analysis*. Principal component analysis is a statistical approach that can be used to analyze the interrelationship among a large number of variables. Here, the information contained in a number of original variables is condensed into a smaller set of variates (factors) with minimum loss of information [10]. This data summarization helps identify the underlying dimension or factor. It also estimates factors and contribution of each variable to the factors (termed loadings).

Suppose we have a set of *N* = 7 variables, *a∗*1*j* to *a∗Nj*, representing the seven COVID-19 variables for each day *j*. Furthermore, let us standardize each variable by its mean and standard deviation: for example,  *a*_*ij*_=(*a*_1*j*_^*∗*^ − *a*_1_^*∗*^)/*s*_1_^*∗*^, where *a*_1_^*∗*^ is the mean of *a*_1*j*_^*∗*^ across days and *s*_1_^*∗*^ is its standard deviation. These selected variables are expressed as a linear combination of a set of underlying components for each day *j*:

(4) $$\begin{array}{c} a_{1j}^{*}=v_{11}*A_{1j}+v_{12}*A_{2j}+,\ldots ,+v_{1N}*A_{Nj},\\ \vdots \\ a_{Nj}^{*}=v_{N1}*A_{1j}+v_{N2}*A_{2j}+,\ldots ,+v_{NN}*A_{Nj}, \end{array}$$

where *j*=1,…, *J*; here, *J*=89*A*′*s* are the components and *v* ′*s* are the coefficients on each component for each variable. The “scoring factors” from the model are recovered by inverting the system implied by (4) and yield a set of estimates for each of the N principal components:

(5) $$\begin{array}{c} A_{1j}=f_{11}*a_{1j}+f_{12}*a_{2j}+,\ldots ,+f_{1N}*a_{Nj},\\ \vdots \\ A_{Nj}=f_{N1}*a_{1j}+f_{N2}*a_{2j}+,\ldots ,+f_{NN}*a_{Nj},\\ j=1,\ldots ,J. \end{array}$$

With respect to this study, there are 7 variables and 89 days. So, *N* = 7 and *J* = 89.

## 3. Results and Discussion

The entire dataset from 22 May to 2 September is classified weekwise. The lockdown was imposed on March 24, 2020. New cases exceeded 30 for the first time on May 22. On this day, total deaths were only 3 and total confirmed cases were 457. The information on the severity of lockdown weekwise is given in Table 1. The controls I, II, III, and IV were exercised by the Government of Nepal to control the spread of the pandemic.

**Figure 1:**
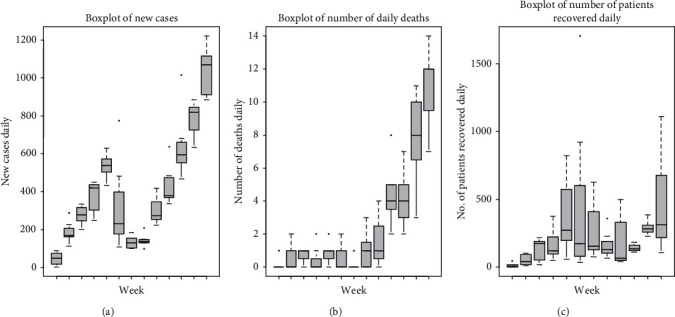
Boxplot of weekly progression of COVID-19 from May 22 to September 2, 2020.

**Figure 2:**
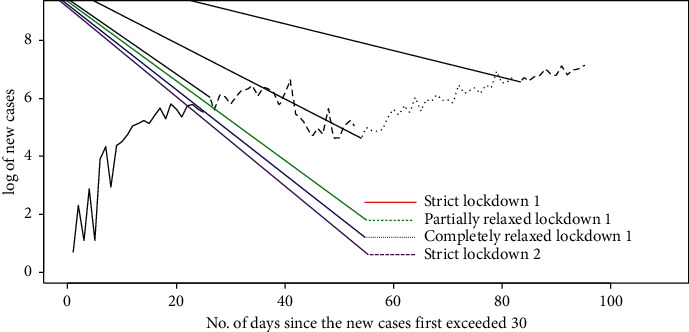
Progression of new cases of COVID-19 over the periods of four controls.

The incidence of daily new cases, daily deaths, and daily recovered is visually displayed by boxplots in Figure 1. Thirteen weeks from May 22 to 2 September are represented by 13 boxplots. As seen from Table 1, during this time, four types of governmental controls were exercised. It can be seen from Figure 1 that the number of new cases and daily deaths is low during week 1–week 3. The smaller size of the box indicates low variance. This is during the period of controls I and II. This number reaches its minimum on week 8. Week 8 is the culmination of the lockdown period and the start of complete relaxation of lockdown, which is control III. In this period of completely relaxed lockdown 1 (control III), there is a steady increase in the number of recovered and daily deaths. This period continues for three weeks. After week 8, the number of new cases and daily deaths rises steadily. This behavior is explained by the influx of workers from India. The number of patients recovered daily increases from week 5. It can be seen from Figure 2 that there is a steady increase in the incidence of COVD-19 cases. The curve starting on May 24 in control I reaches a low value on 22 July 2020 in control III. It is the culmination of lockdown and start of the period of complete relaxation. Thereafter, the number of COVID-19 infected increases steadily.

**Figure 3:**
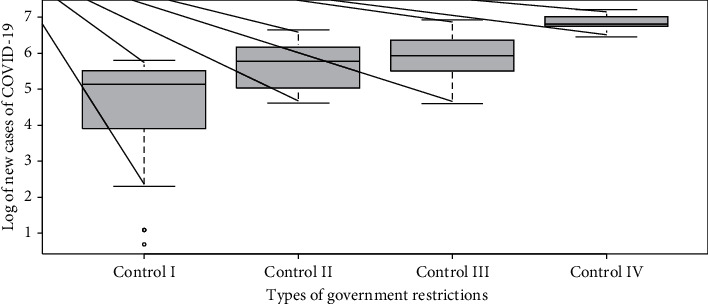
Boxplot of incidence of new cases during the period of control.

The change in the pattern of occurrence of new cases of COVID-19 over progression of time is also exhibited in boxplots of Figure 3. It is seen that, in the period of strict lockdown 2 (control IV), the incidence of COVID-19 cases are very high and the variance among number of cases is the lowest. In contrast to this, the period of strict lockdown 1 and partially relaxed lockdown 1, controls I and II, has low incidences with a high variance.

The descriptive statistics of variables related to COVID-19 is provided in Table 2. Time using series analysis is performed here using ARIMA models. ARIMA models are suitable for explaining the overall trend of the data with seasonal fluctuations. This pattern is exhibited by the COVID-19 data of Nepal. But, when there are sudden spikes or drops in the data due to outlier values, ARIMA models should be used with care. The average number of new cases per day is 412 with a standard deviation of 287.198. The average number of daily recovered per day is 217, and the standard deviation is 270.801. The average number of daily deaths is 3 with a standard deviation is 3.541. The average number of total isolated is 7325, and the standard deviation is 4387.101. It can also be seen from Table 2 that the time series progression of log of new cases can be explained with an ARIMA (0, 1, 2) with an AIC of 69.72. Here, log stands for log to the base *e*. Similarly, log of total isolated and total deaths can be illustrated by ARIMA (0, 2, 1) and ARIMA (0, 2, 2). The AIC are −249.17 and −229.67, respectively. The smaller the value of AIC, the better the model in explaining these scenarios related to COVID-19. Here, ARIMA stands for autoregressive integrated moving averages. Similarly, daily deaths can be explained by ARIMA (0, 1, 1) with an AIC of 348.06 and daily recovered by ARIMA (0, 0, 1) with an AIC of 825.2.

**Figure 4:**
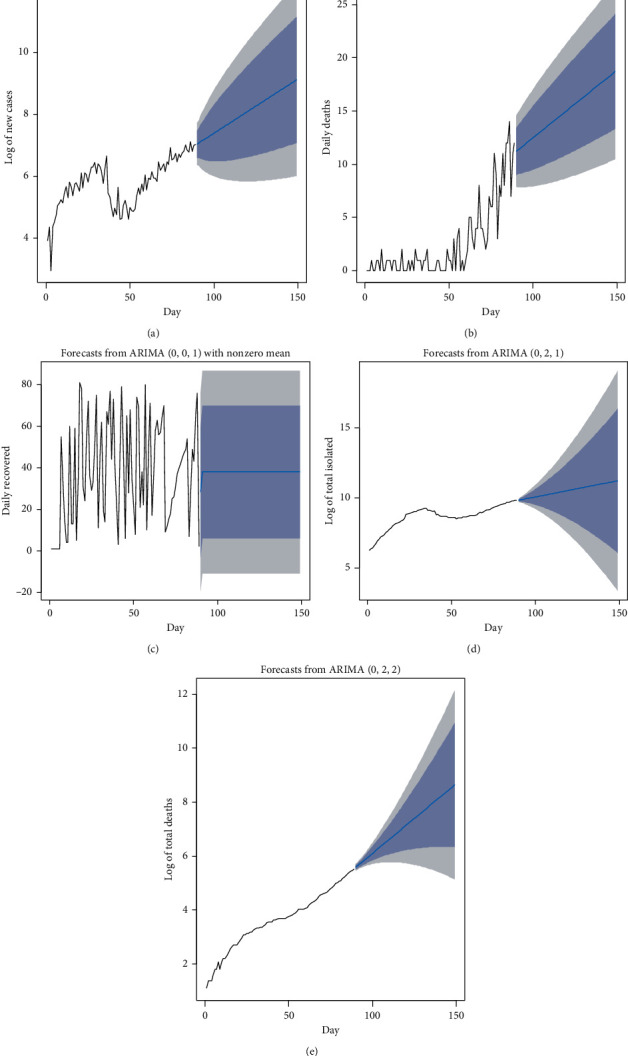
The predicted values for the next 60 days.

The predicted values for another 60 days in the future can be seen in Figure 4. These are based on ARIMA values given in Table 2. The trend of the values forecasted into the future is seen in this figure.

**Figure 5:**
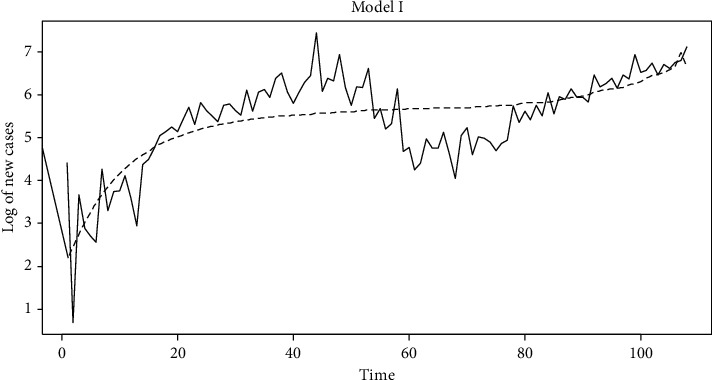
Comparison between the observed and predicted values for model I.

**Figure 6:**
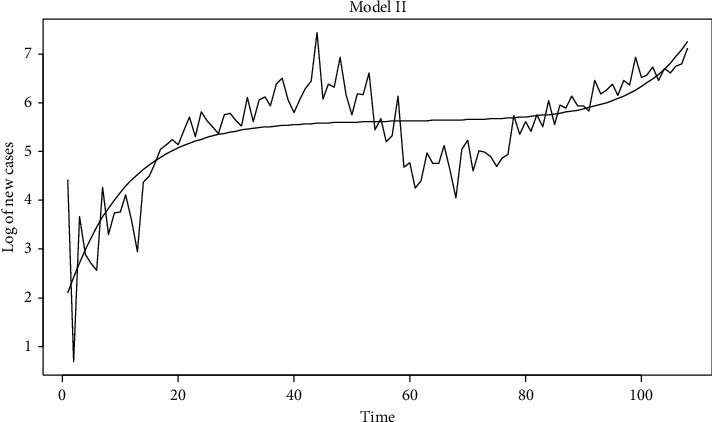
Comparison between the observed and predicted Values for model II.

Model I and model II explain the incidence of new cases using nonlinear regression. The results are given in Table 3. As seen from this table, in model I, the incidence of new cases of COVID-19 is explained by regressing it on the day and total deaths. Here, the total death value available from any previous time, preferably near to the day, is given as the input. The accuracy of the model is 99.99936%. Similarly, in model II, the incidence of new cases of COVID is regressed on day. The accuracy of this model is 99.99945%. As seen from Table 3, the Shapiro–Wilk test for normality, under the null hypothesis that the data are normally distributed, gave a *p* value of 0.046 and 0.043. These are from model I and model II, respectively. In Table 3, the regression coefficients are said to be very highly significant if the *p* value is equal to 0. Similarly, if the *p* value is less than 0.01, then it is said to be significant. Close correspondence between observed and predicted values are also shown in Figures 5 and 6.

New cases, deaths, recovered, and total isolated are classified into seven categories. Total deaths are classified into six categories. The seven categories of new cases are 0–30, 30–50, 50–100, 100–250, 250–500, 500–800, and 800–1200. The seven categories of deaths are 0–2, 2–4, 4–6, 6–8, 8–10, 10–12, and 12–15. The seven categories of recovered are 0–245, 245–490, 490–735, 735–980, 980–1225, 1225–1470, and 1470–1715. Total isolated are classified into 0–1000, 1000–3000, 3000–6000, 6000–10000, 10000–15000, 15000–21000, and 21000–27000. Finally, total deaths are divided into 0–5, 5–15, 15–35, 35–75, 75–155, and 155–315. These categories are used in multinomial logistic regression, odds ratio, and linear mixed-effect models, elucidated below.

The sensitivity to change in new cases with change in total deaths and week is explained with multinomial logistic regression. This model explains 61.7% of the total variance as *R*^2^=0.617. The behavior of odds ratio is displayed in Table 4. As seen from this table, odds in favor for daily new cases to rise from 0–30 to 100–250 increases by 1408 times as total deaths mount by 1 category. Also, it increases by 2168 and 3170 times for a rise in new cases from 0–30 to 500–800 and 800–1200, respectively. Similarly, odds in favor for daily new cases to rise from 0–30 to 50–100 increases by 978.526 times as weeks mount by 1 unit. Also, it increases by 2281 times for a rise in new cases from 0–30 to 800–1200. This implies that, as total deaths increase and weeks progress, under current situation, the chances that the daily new cases increase to 800–1200 are the highest. These are all statistically very highly significant with *p* values less than 0.01. It is also seen that odds in favor for the incidence of new cases to rise from 0–30 to 1200–1500 increases by several times. This trend is validated by the data from September 3 to the current date of 13 September.

**Figure 7:**
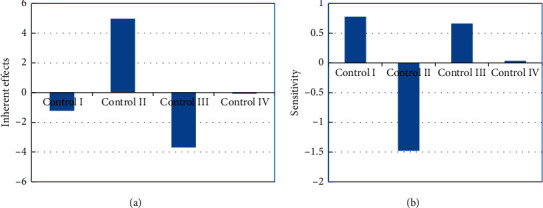
The inherent effect (intercept) and the sensitivity (slope) of the random effects.

Linear mixed-effect models are used in quantifying the random effect of control imposed by the Government of Nepal. These controls are categorized as strict lockdown 1, partially relaxed lockdown 1, completely relaxed lockdown 1, and strict lockdown 2. This sheds light on the behavior of new cases with impact of total deaths and weeks as fixed effect and control as random effect. The data are nested in control. As seen from Table 5, this classification into fixed effects and random effects explains 97.557% of the error variance. This nesting of the data under the variable control explains 90.452% of the error variance. As seen from random-effect heading, the intrinsic impact of control II, which is partially relaxed lockdown 1, is the highest, as the intercept is 4.966. The sensitivity of incidence of new cases is also the highest. This is highlighted by the slope equal to −1.477. This further implies that, with one unit increase in total death, the incidence of new cases decreases by 1.477. The period of completely relaxed lockdown 1 shows the intrinsic effect of −3.685 with a sensitivity of 0.664. Slope and intercept of a regression equation can be physically interpreted as sensitivity and inherent effect, respectively. This effect is visually displayed in Figure 7. As seen from this figure, the period of control II has highest inherent effects and sensitivity. It is seen that the inherent random effects of total deaths on the incidence of new cases are highest in control II and control III. These are the periods between two strict lockdowns. Similarly, the sensitivity is also highest in these periods. This is true as control II and control III, as also seen from Table 1, are time periods of partially relaxed lockdown 1 and completely relaxed lockdown 1. So, in this period, governmental controls are partially and completely relaxed, resulting in more interactions among people. There was a spike in new cases in these periods.

The principal component analysis of the seven variables, namely, new cases, daily deaths, daily recovered, total cases, total recovered, total infected (isolated), and total deaths gives one principal component. This component explains 74.579 percent of the total variances. Thus, dimensionality of the data comprising seven interrelated variables is reduced by one variable. The factor scores of the first principal component on daily total deaths are the highest and amount to 0.979. Daily total cases, daily total infected, daily deaths, daily new cases, and daily total recovered are 0.955, 0.926, 0.881, 0.859, and 0.851. The factor score is the lowest for daily recovered and is 0.511. In Nepalese context, it is very difficult to get accurate records of daily recovered as many COVID-19 patients are in home quarantine. The hospitals for COVID-19 are not accessible socially and economically to certain sections of the society. Thus, the values of daily recovered might not be completely accurate. So, the first principal component can be taken as an indicator of accurate data, as it has high factor scores on new cases, daily deaths, total cases, total recovered, total infected (isolated), and total deaths. It can be said to be linked with the variables with accurate values as it has higher factor scores on these variables.

As the World Health Organization is intervening in the eradication of this pandemic in the whole world including Nepal, first principal component can be taken as the representative of variables of higher accuracy. The dimension of the seven variables, new cases, daily deaths, daily recovered, total cases, total recovered, total infected (isolated), and total deaths, is reduced to one principal component. This principal component explains 74.579 percent of total variation.

At present, India has become the epicenter of COVID-19 with daily new cases becoming the highest in the world. The new cases of COVID-19 infected in India on 13 September were 93,215, which is the highest in the world [11]. It surpasses the United States and Brazil with the incidence of 31,857 and 14,597, respectively. With the easing of strict lockdown 2 to partially relaxed lockdown 2 on September 10, 2020, the number of COVID-19 cases in Nepal could increase drastically. Nepal shares an open border with India, and people can easily cross the border without any legal documents and do not require any work permit to work on either side of the border. Many Nepalese work abroad, including India [12]. The positive cases of COVID-19 are either Indian nationals working in Nepal or Nepali workers who have recently returned from India. Thousands of workers who have returned to Nepal without proper screening are from the Indian states of Maharashtra, Delhi, and Gujarat. Many workers are stranded in different parts of India due to lockdowns in both countries. Many workers are stranded in the border [13–17]. They could be the potential cases of COVID-19, upon their return to Nepal. The spread of the disease can be controlled if the public follows the guideline provided by the Ministry of Health and Population, Government of Nepal [18]. Although the Government of Nepal implemented early school closure and lockdown, the management to contain COVID-19 does not seem to be sufficient. Understanding the current situation regarding COVID-19 in Nepal is important for providing a direction towards proper management of the disease [19].

## 4. Conclusion

Direct changes are obvious and can be easily measured. But, in occurrence of such a pandemic, inherent variables need to be identified and measured. This paper studied these variables and their interrelationships. But, such variables cannot be directly measured, especially in countries with a background limited and scarce data. This analysis is also performed from the perspective of four controls exercised by the Government of Nepal. They are as follows: Control I is strict lockdown 1, from March 24 to June 11, 2020. Control II is from June 11 to July 21 and is called partially relaxed lockdown 1. Control III is from July 21 to August 19 and is called completely relaxed lockdown 1. The period from August 19 to September 2 is called strict lockdown 2 and is classified as control IV.

Here, data are also classified into categories, and the impact on the new cases is studied using multinomial logistic regression and odds ratio. Principal component analysis is used to study inherent interrelationships between the variables.

Two nonlinear models explaining the incidence of new cases in Nepal are also explained. These models have given very promising results for India as well. This highlights the robustness of these models. It can be used in explaining the incidence of COVID in South Asia.

It is also seen that change in government controls from strict lockdown to partially relaxed lockdown and completely relaxed lockdown and then strict lockdown has a detrimental effect. The incidence of daily new cases increases during this change of the government control system. The change from one control system to another does not seem to control the spread of the pandemic. So, it is advisable to exercise one system of governmental control. The public should adjust to that system and engage in their day-to-day personal and professional activities under that one system. This will help in controlling the spread of the pandemic.

The incidence of this disease over a period of one year will be studied in the future. A detailed household survey on the pattern of incidence of COVID-19 is also planned as future work.

## Figures and Tables

**Table 1:** Governmental restriction to control the spread of COVID-19.

Week	Dates	Severity of lockdown	Control
1. Week 1–week 3	22 May 2020 to 11 June 2020	Strict lockdown 1	I
2. Week 4–week 7	12 June 2020 to 21 July 2020	Partially relaxed lockdown 1	II
3. Week 8–week 11	22 July 2020 to 19 August 2020	Completely relaxed lockdown 1	III
4. Week 12–week 13	20 August 2020 to 2 September 2020	Strict lockdown 2	IV

**Table 2:** Details of the variables considered for this study.

Sr. no.	Variables	Min	Max	Mean	Median	Mode	SD	ARIMA	AIC	Categories
1	New cases (daily)	19	1221	412	331	150	287.198	ln-ARIMA(0, 1, 2)	69.72	1, 2, 3, 4, 5, 6,7
2	Deaths (daily)	0	14	3	1	0	3.541	ARIMA(0, 1, 1)	348.06	1, 2, 3, 4, 5, 6, 7
3	Recovered (daily)	0	1705	217	134	0	270.801	ARIMA(0, 0, 1)	825.2	1, 2, 3,4, 5, 6, 7
4	Total isolated	513	18112	7325	6544	-	4387.101	ln-ARIMA(0, 2, 1)	-249.17	1, 2, 3, 4, 5, 6, 7
5	Total deaths	3	251	62	40	40	61.510	ln-ARIMA(0, 2, 2)	-229.67	1, 2, 3, 4, 5, 6

**Table 3:** Details of two best models explaining the evolution COVID-19 in Nepal.

Sr. no.	Model	*R* ^2^	Shapiro–Wilk test *P* value
1	Log(new cases daily)=*a* − (*b*/2)*e*^−*c∗*Day^+*d∗*total deaths	0.9999936	0.04601
2	*a* = 5.418^*∗∗*^, *b* = 7.30^*∗∗*^, *c* = 0.104^*∗∗*^, and *d* = 0.007^*∗∗*^	0.9999945	0.04314
	Log(new cases daily)=*a* − (*b*/2)*e*^−*c∗*Day^+(*d*/2)*e*^*e∗*Day^		
	*a* = 5.628^*∗∗*^, *b* = 7.781^*∗∗*^, *c* = 0.0973^*∗∗*^, *d* = 0.000038, and *e* = 0.105^*∗*^		

^*∗∗*^Very highly significant; ^*∗*^significant. Total deaths are from some previous days.

**Table 4:** Odds in favor for new cases to rise from 0 to 30.

New cases	Total deaths	Week
30–50	0^*∗∗*^	0^*∗∗*^
50–100	1.937^*∗∗*^	978.526^*∗∗*^
100–250	1408.185^*∗∗*^	150.981^*∗∗*^
250–500	392.482^*∗∗*^	249.977^*∗∗*^
500–800	2168.014^*∗∗*^	196.192^*∗∗*^
800–1200	3170.155^*∗∗*^	2281.025^*∗∗*^

^*∗∗*^Very highly significant.

**Table 5:** Results of the linear mixed-effect model of new cases.

Model	Percent	Fixed effect	New cases = 2.845+0.232^*∗*^ total deaths+ 0.198^*∗*^week	Final form = fixed effect + random effect
1. *R*^2^	75.6	Random effect	Control 1: −1.215+0.777^*∗*^ total deaths	Control 1: new cases=1.630+1.010^*∗*^ total deaths +0.198^*∗*^ week
2. Total error variance explained	97.557		Control 2: 4.9656 − 1.477^*∗*^ total deaths	Control 2: new cases=7.810 − 1.245^*∗*^ total deaths +0.198^*∗*^ week
3. Total error variance explained by nesting	90.452		Control 3: −3.685+0.664^*∗*^total deaths	Control 3: new cases=−0.840+0.896^*∗*^ total deaths +0.198^*∗*^ week
			Control 4: −0.066+0.036^*∗*^total deaths	Control 4: new cases=2.779+0.269^*∗*^ total deaths +0.198^*∗*^ week

## Data Availability

The COVID-19 updates data from the Ministry of Health and Population, Government of Nepal, were used in this study. COVID-19 updates of the WHO and Our World in Data-COVID-19 were used for validation.
